# Ethanol inducible expression of a mesophilic cellulase avoids adverse effects on plant development

**DOI:** 10.1186/1754-6834-6-53

**Published:** 2013-04-16

**Authors:** Holger Klose, Markus Günl, Björn Usadel, Rainer Fischer, Ulrich Commandeur

**Affiliations:** 1 Institute for Molecular Biotechnology (Biology VII), RWTH Aachen University, Worringerweg 1, Aachen, 52074, Germany; 2 Institute of Bio- and Geosciences, IBG-2: Plant Sciences, Forschungszentrum Jülich, Leo-Brandt-Straße, Jülich, 52425, Germany; 3 Institute of Biology I, RWTH Aachen University, Worringerweg 1, Aachen, 52074, Germany; 4 Fraunhofer Institute for Molecular Biology and Applied Ecology (IME), Forckenbeckstrasse 6, Aachen, 52074, Germany

**Keywords:** Cellulose, Recombinant cellulase, Transgenic plant, Chemically inducible expression, Cell wall degradation, *Trichoderma reesei*, *Nicotiana tabacum*

## Abstract

**Background:**

Plant-produced biomass-degrading enzymes are promising tools for the processing of lignocellulose to fermentable sugars. A major limitation of *in planta* production is that high-level expression of such enzymes could potentially affect the structure and integrity of the plant cell wall and negatively influence plant growth and development.

**Results:**

Here, we evaluate the impact on tobacco plant development of constitutive versus alcohol-inducible expression of the endoglucanase TrCel5A from the mesophilic fungus *Trichoderma reesei*. Using this system, we are able to demonstrate that constitutive expression of the enzyme, controlled by the doubled Cauliflower Mosaic Virus promoter, leads to lower cellulose content of the plant combined with severe effects on plant growth. However, using an alcohol-inducible expression of the endoglucanase in the plant leaves, we achieved similar enzymatic expression levels with no changes in the crystalline cellulose content.

**Conclusion:**

We were able to produce significant amounts of cellulase in the plant leaves without detrimental effects to plant development. These results demonstrate the potential feasibility of an inducible expression system for producing biomass degrading enzymes in plants.

## Background

Plant biomass is a promising alternative to conventional, non-renewable sources of energy. In first generation biofuels, easily fermentable carbon sources such as starch and sucrose have primarily been used. An alternative approach is to convert lignocellulosic biomass into fuels or platform chemicals. The process required for this is much more complex than the fermentation of simple carbohydrates and one of the bottlenecks is the short supply of biomass-degrading enzymes, such as cellulases. Plants can express specific recombinant proteins [1-3], so the heterologous production of lignocellulolytic enzymes in plants could help to process biomass more efficiently [4-6].

Cellulases and other glucanases break down the plant cell wall [7,8] and also play a role in cell wall development and remodeling [9-11]. It is therefore necessary to consider the potential impact of heterologous cellulases on normal plant growth and development. This is particularly relevant when using strong promoters like the *Cauliflower mosaic virus* (CaMV) 35S promoter derived from dicotyledons [12], the tissue specific maize embryo-preferred globulin-1 promoter [13] or the artificial Mac promoter [14], based on the *Escherichia coli* maltose and lactose operons [15].

The high-level constitutive expression of glucanases has frequently been shown to affect the structure and composition of plant cell walls by altering the quality and/or quantity of cellulose. For example, the constitutive overexpression of an aspen endoglucanase (PttCel9A1) in *Arabidopsis thaliana* reduced cellulose crystallinity and the glucose content in the cell wall [10]. Glucanase overexpression can also modify leaf shape and growth [16-18] and reduce environmental stress tolerance [4]. Potential mitigation strategies include the localization of glucanases in subcellular compartments, such as the endoplasmic reticulum (ER) and the storage vacuoles or plastids [12,13,17], and the expression of glucanases that are inactive at physiological temperatures but inducible using a post-harvest temperature shift [19-22].

In contrast to constitutive expression techniques, we investigated the regulation of cellulase expression in tobacco using an alcohol-inducible promoter. Many different inducible expression systems have been developed for plants [23-26] and the *Aspergillus nidulans* alc platform has been widely used [27-29]. This platform comprises two elements, the coding sequence for the transcription factor AlcR (controlled by the CaMV 35S promoter), and the *alcA*min 35S promoter, in which two AlcR binding sites are joined to the CaMV 35S minimal promoter [27]. This promoter shows negligible basal expression and a strong induction ratio in most plants [27,28,30]. We used the *alc* platform to inducibly express a mesophilic endoglucanase (TrCel5A) from the filamentous fungus *Trichoderma reesei*, the second most abundant of eight endoglucanases secreted by this species [31,32].

## Results

### Cloning TrCel5A and the generation of transgenic tobacco plants

**Figure 1:**
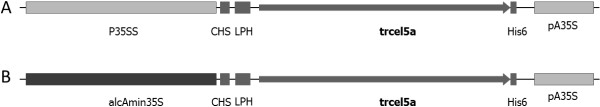
**Schematic presentation of the plant expression cassettes for constitutive (A) and inducible (B) expression of TrCel5A.** The CaMV promoter (P35SS) and terminator signal (pA35S) are indicated in light grey. The chimeric promoter (alcAmin35S) comprising the CaMV 35S minimal promoter (±31 to +5) fused to the upstream promoter sequences of *alc*A (Caddick et al., 1998) is shown in black. 5′-UTR of chalcone synthase (CHS), plant codon-optimized leader peptide (LPH) derived from the heavy chain of the murine mAb24, the gene of interest (trcel5A) and the His_6_ coding sequence (His6) are indicated in dark grey.

The cDNA encoding TrCel5A (without its native signal peptide sequence) was introduced into two different plant expression vectors, one under the control of the constitutive double CaMV 35SS promoter and the other under the control of the inducible *alcA*min35S promoter [27]. Both constructs included a plant codon-optimized leader peptide, derived from the heavy chain of the murine monoclonal antibody mAb24 for secretion to the apoplast, and a C-terminal His_6_ tag for detection and purification (Figure 1). The functionality of the cloned constructs was verified by transient expression [33] followed by the detection of the active enzyme by Western blot and activity staining (Additional file 1).

Both constructs were introduced into tobacco (*Nicotiana tabacum* SR1) leaf by *Agrobacterium*-mediated transformation [34]. Each generation of plants was screened for enzyme expression by Western blot and activity staining using azocarboxymethylcellulose (azoCMC) and 4-methylumbelliferyl-β-cellobiose (4-MUC). Positively tested T1 lines showing 3: 1 segregation, consistent with a single locus insertion, were then used to produce homozygous T2 lines. These T2 lines were characterized as follows.

### TrCel5A expression in tobacco plants

**Figure 2:**
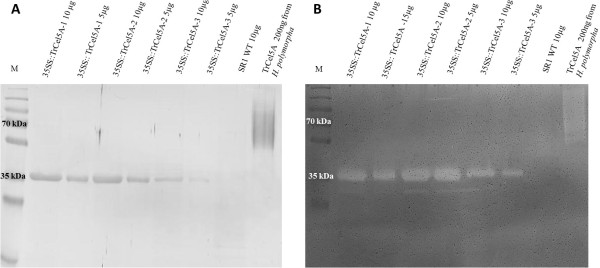
**Western blot of different transgenic lines constitutively expressing TrCel5A after SDS-PAGE (A) and zymography performed with SDS-PAGE containing 0.15% (w/v) CMC (B).** Lanes contain 10 μg of plant total soluble protein. The recombinant enzyme was detected with a polyclonal α-cellulase antibody and an alkaline phosphatase conjugated goat-anti-rabbit secondary antibody. The zymogram control is performed with purified TrCel5A produced in *Hansenula polymorpha*.

Western blot analysis showed the recombinant protein had a molecular weight of approximately 35 kDa (Figure 2A), which is similar to the molecular weight of the native catalytic domain [35]. The truncated enzyme retained its activity (Figure 2B). TrCel5A expression levels in transgenic tobacco plants were determined by measuring the activity of total soluble protein (TSP) against the substrates azoCMC and 4-MUC. Plants with constitutive TrCel5A expression achieved a specific enzyme activity of up to 1.5 U mg^-1^ TSP for azoCMC and 35 nmol 4MU min^-1^ mg^-1^.

**Figure 3:**
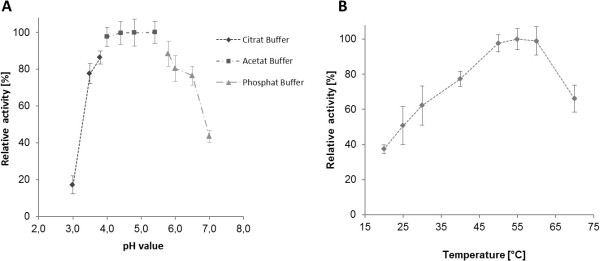
**Enzymatic activity of TrCel5A at different pH and temperature values using the azoCMC assay.** Activity of TrCel5A was determined for pH values between 3.0 and 7.0 (**A**) and temperature between 20–70°C (**B**). The maximum activity measured for each system (at pH 4.8 and 55°C, respectively) was set to 100%. For panel A, the buffer systems are as indicated in the figure and temperature was set at 55°C. For panel B 50 mM Na-Acetate, pH 4.8 buffer was used throughout.

The biochemical properties of purified TrCel5A were tested on azoCMC, within the pH range 3.0–7.0 and the temperature range 20–70°C (Figure 3). The optimal conditions were pH 5.4 and 55°C, at which 50% of the enzyme remained active for 90 min. There was no significant activity against highly crystalline substrates, such as avicel, however we detected significant activity against heterogeneous substrates including lichenan and barley-β-glucan (Table 1).

**Table 1:** Hydrolytic activity of TrCel5A on different polymeric carbohydrates

	**CMC**	**Barley β-glucan**	**Lichenan**	**PASC**	**Avicel**	**Oat-Xylan**
**Relative activity [%]**	100±2	120±3	136±5	22±2	N.D.	N.D.

The amount of released reducing sugars was determined using a PAHBAH assay. Activity against carboxymethylcellulose (CMC) was set as 100%. N.D indicates non detectable values.

### Inducible TrCel5A expression in tobacco plants

**Figure 4:**
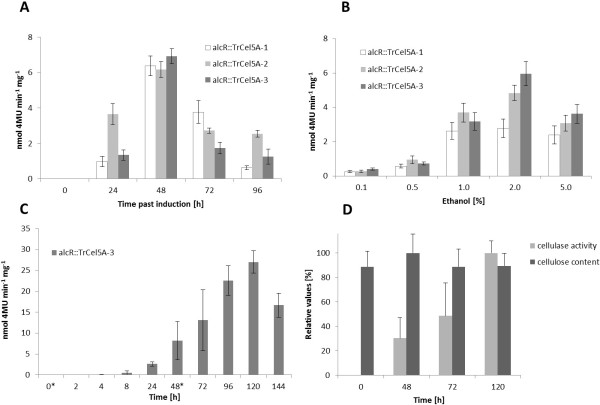
**Effects of Ethanol induction on transgenic plants.** Activity on 4MUC measured over time after ethanol induction in six-week-old soil-grown plants (**A**). Plants were induced at t0 by applying 2% ethanol in 100 ml irrigation water, and cellulase activity was monitored over a 96 h time course. Ethanol dose response in alcR::TrCel5A lines (**B**). Transgenic lines were monitored for cellulase activity 24 h after watering. Values represent the mean of three plants per independent transgenic line. Effect of sequential ethanol induction (**C**). Six-week-old soil-grown plants from the homozygous line F6.5 were induced using 2% ethanol at t0 and again after 48 h (asterisks). Cellulase activity was monitored over 144 h. Wild-type plants were monitored in parallel, and no cellulase activity was observed throughout the time course. Comparison of cellulose content and cellulase activity after repeated induction with ethanol (**D**). A relative value of 100% cellulase activity represents a conversion of 27 nmol 4MU min^-1^ mg^-1^, whereas a value of 100% for cellulose content represents 140 μg glucose per mg alcohol insoluble residue (AIR). For all panels, values represent the mean of three plants per independent transgenic line. Error bars the show standard deviation of the mean after subtraction of wild-type control data.

We examined the effects of alcohol induction on plant growth and TrCel5A expression. In all assays T2 TrCel5A plants were compared to wild-type plants. Initial induction of TrCel5A activity using 2% ethanol led to a maximum expression level of 0.4 U mg^-1^ TSP on azoCMC and 5 nmol 4MU min-^-1^ mg-^-1^ after 24 h (Figure 4A). Further analysis showed that significant induction could be achieved with 0.1% ethanol (without basal expression) but that optimal expression levels required 2% ethanol (Figure 4B). There was no improvement in cellulase expression in transgenic plants when 5% ethanol was used, and concentrations greater than 5% were detrimental to both wild-type and transgenic plants, resulting in physiological stress symptoms such as leaf yellowing and curling (data not shown).

To determine the time course of induction, cellulase activity was monitored for 4 days following the induction with 2% ethanol (Figure 4A). Peak activity of 7 nmol 4MU min^-1^ mg^-1^ was observed 48 h after induction, followed by a significant decline. Continuous or sequential induction can increase transgene expression levels considerably [28], so expression was induced again 48 h after the first induction, increasing the activity significantly to 27 nmol 4MU min^-1^ mg^-1^ (Figure 4C). Although this activity level was lower than that achieved with constitutive expression, the two-step induction nevertheless achieves an expression level in the same order of magnitude as the constitutive promoter.

### Growth characteristics and histology

**Figure 5:**
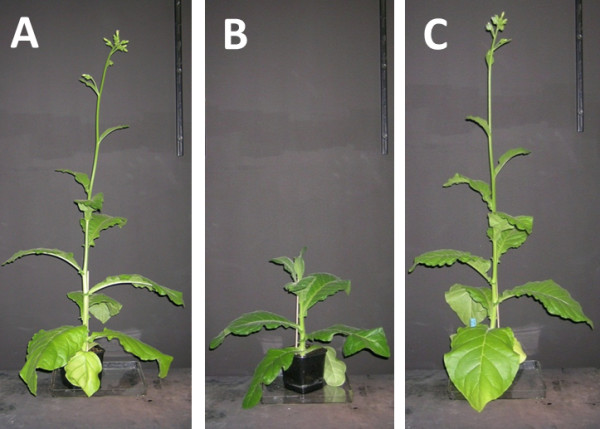
**Phenotype of transgenic tobacco plants.** Strains shown are wild-type *N. tabacum* SR1 (**A**). and transgenic tobacco strains with constitutive TrCel5A expression 35::TrCel5A (**B**). and inducible TrCel5A expression alcR::TrCel5A (**C**). Plants were grown under photoautotrophic conditions in soil. Images shown are representative of three plants per genetic line.

**Figure 6:**
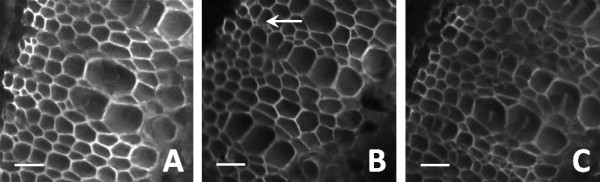
**Cross sections of transgenic and wild-type tobacco stems (imaged at 10x) stained with calcofluor-white and visualized under UV light.** Tissue sections of wild-type plants (**A**) were compared with 35SS::Trcel5A (**B**) and alcR::TrCel5A (**C**) transgenic plants. No significant difference was detected between the wild-type and transgenic plants, except a marginal increase in the number of small vessels (white arrow) in 35SS::Trcel5A plants. Scale bars are 50 μm.

Constitutive TrCel5A expression significantly reduced the growth of tobacco plants and delayed their development compared to wild-type. The mature stems of the transgenic lines were 21–36% shorter than control plants and flowering was delayed (Figure 5). Cross-sections of stems, stained with calcofluor-white, showed no significant difference in cell structure between transgenic T2 lines constitutively expressing TrCel5A and SR1 wild-type plants, although a marginally greater number of small vessels were apparent (Figure 6). In contrast to constitutive expression, inducible TrCel5A expression had no impact on the growth or development of tobacco plants (Figure 5).

### Chemical analysis of tobacco cell walls

The impact of TrCel5A expression on the structure of tobacco cell walls was determined, specifically the amount of cellulose and alcohol-insoluble residues (AIRs) were quantified as described previously [36]. Additionally, the crystalline cellulose content was measured by Updegraff hydrolysis followed by and an anthrone cellulose assay [37]. In all assays, wild-type plants were compared with three constitutive transgenic lines and two inducible transgenic lines expressing TrCel5A.

The constitutive transgenic lines contained significantly lower levels of crystalline cellulose than the wild type plants (Table 2). The transgenic line 35SS::TrCel5A-2 displayed the highest enzyme activity and also showed the greatest reduction in cellulose. In contrast, there was no difference in crystalline cellulose levels between inducible transgenic plants and wild-types, even after the induction of cellulase activity (Figure 3D).

**Table 2:** Content of crystalline cellulose in leaves of 6-week-old soil grown transgenic T2 plants compared to wild-type tobacco plants

	**SR1 wild-type**	**35SS::**	**35SS::**	**35SS::**	**alcR::**	**alcR::**
		**TrCel5A-1**	**TrCel5A-2**	**TrCel5A-3**	**TrCel5A-2**	**TrCel5A-3**
**Cellulose [μg Glc per mg AIR]**	148.86 ± 10.72	118.42* ± 6.33	95.40* ± 11.90	117.50* ± 12.80	147.60 ± 11.18	135.58 ± 13.96

Values are the mean ± standard error of the mean of 10 plants per tobacco line (transgenic or wild-type). Asterisks indicate a significant difference compared to the wild-type (P-value ≤ 0.01).

## Discussion

The exact role of glucanases in the development of plants and their cell wall is not yet fully understood. Both the absence and overabundance of glucanases has an impact on plant growth and cell wall development, which is a major challenge for the expression of recombinant glucanases in plants for biomass degradation [9,10].

Plants have been widely used for the production of recombinant proteins [1,2] including glucanases and other biomass-degrading enzymes [4,6]. However, in many instances the constitutive expression of such enzymes has been shown to negatively affect plant health, including reducing plant growth, altering the morphology of leaves, reducing plants’ stress tolerance or changing the structure of the cell wall [4,16,17,21,38]. Alternative strategies are therefore desired for the production of cellulases in plants without affecting growth or development. Some suggested strategies include sequestration into subcellular compartments [14] and the expression of enzymes that are inactive under physiological conditions [19]. We have shown that inducible promoter systems can also be used to successfully produce cellulases without impacting on plant development.

Cellulases from the mesophilic fungus *T. reesei* efficiently degrade plant biomass. The endoglucanase TrCel5A (formerly EGII or EGIII) comprises a C-terminal catalytic domain from glycoside hydrolase family 5, a Ser/Pro-rich linker and a family 1 carbohydrate-binding module (CBM) [39]. We used two different promoters to express TrCel5A, in each case combined with a signal peptide to achieve targeting to the apoplast. The double CaMV 35S promoter was use for constitutive expression, and its effects on plant growth and development were compared to those of the ethanol-inducible *alcA*min35S promoter. Transient expression revealed the proteolytic cleavage of TrCel5A within the linker region (Additional file 1), probably caused by papain-like proteases in the apoplast [40] that are known to degrade *T. reesei* cellulases [41]. The truncated enzyme remains active against soluble substrates, such as azoCMC and other β-glucans with a β(1-4)-linked glucose backbone, whereas carbohydrates with different linkages remain unaffected [35]. The optimal reaction conditions were demonstrated to be 55°C and pH 4.8, highlighting the mesophilic origin of the enzyme. However, the enzyme also showed remarkable residual activity under physiological conditions (20–30°C, pH 5.0), suggesting it could interfere with the synthesis of cell wall cellulose during normal plant growth and development.

TrCel5A activity in constitutive transgenic plants was determined by measuring the conversion of 4MUC into 4MU. The highest expression level observed was 35 nmol 4MU min^-1^ mg^-1^, which is similar to previously reported values for *Acidothermus cellulolyticus* E1 [14,21]. The transgenic plants grew more slowly than their wild-type counterparts, and they contained lower levels of crystalline cellulose. It is unclear whether the reduced cellulose content reflected only slower, more prolonged growth of the plants (i.e. where the plants would eventually achieve the same biomass as the wild-type plants) or whether, as suggested by the shorter stems in transgenic plants, the reduced levels of crystalline cellulose are permanent. Glucanases have previously been shown to affect the cellulose content and hence the growth of transgenic plants, suggesting the same phenomenon was responsible for the phenotype we observed [10].

Inducible expression of cellulases has the potential to allow enzyme expression without detrimental effects on plant growth and development. We therefore chose an ethanol-inducible expression cassette that has previously been used for crop improvement [42]. The one step induction procedure using 2% ethanol had no significant effect on the health of wild type tobacco plants. This induction, when used on alcR::TrCel5A plants resulted in a significant gain in cellulase activity, due to successful induction of TrCel5A. After 24 h this activity was measured to be 4–6 times lower than the TrCel5A activity seen in constitutive transgenic plants. We evaluated ways to increase the cellulase production in the alcR::TrCel5A plants by analyzing the effects of different ethanol concentrations and induction times, and found that for our system 2% was the optimal ethanol concentration for induction, in agreement with previous literature [28]. TrCel5A activity peaked at 7 nmol 4MU min^-1^ mg^-1^ 48 h after induction, which was still significantly lower than achieved in the transgenic plants with constitutive enzyme production, which showed a specific enzyme activity for azoCMC and 35 nmol 4MU min^-1^ mg^-1^. Previous work indicated that multiple inductions can increase transgene expression significantly [28], thus we introduced a second induction step 48 h after the first, which boosted enzyme activity to 27 nmol 4MU min^-1^ mg^-1^ five days after the first induction. Although still lower than the activity seen with constitutive expression, this level of enzyme expression was certainly within the same order of magnitude. Thus inducible TrCel5A can be expressed at levels comparable to that achieved by other researchers [14,21].

The crystalline cellulose content of alcR::TrCel5A plants did not change significantly before and after induction. These data suggest that the endoglucanase only decreases the crystallinity of the cell wall while cellulose is synthesized, such as occurs during constitutive expression. This theory is also supported by the lower endoglucanase activity of TrCel5A against more crystalline substrates, such as Avicel and PASC (Table 1). The apparent loss of the CBM could also play a role in the lower affinity towards crystalline substrates [43]. Alternatively, the efficient hydrolysis of crystalline cellulose may require more than one cellulolytic activity [44]. These data suggest that the absence of the CBM might also be advantageous for systems using constitutive expression of TrCel5A. Potentially, the expression of CBMs alone can disrupt cell wall architecture and interfere with plant development [45] or act as a microbe-associated molecular pattern (MAMP) to trigger a defense reaction, such as the accumulation of sesquiterpene cyclase [46,47].

In summary, we demonstrated that the constitutive expression of the mesophilic endoglucanase TrCel5A has an impact on plant growth and development. We showed that these detrimental effects can be avoided by using the ethanol-inducible promoter *alcA*min35S. Furthermore, sequential induction achieved recombinant cellulase levels similar to constitutive expression. We propose that inducible expression of biomass degrading enzymes e.g. cellulases in plants can be a promising alternative for their *in planta* production. This strategy offers the possibility of controlled application due to different expression conditions of these enzymes e.g. at a certain time point during the plant development or at a defined expression level. In addition, our system utilizes an economically priced inducer and a comparatively simple application able to be applied on a large scale, underline the potential of this approach for biomass degradation.

## Methods

### Construction of plant expression vectors

The TrCel5A gene (EGR51020.1, EMBL-CDS) was amplified by PCR from *T. reesei* strain QM9414 cDNA (kindly provided by Armin Merckelbach, Institute of Molecular Biotechnology, RWTH Aachen University, Germany) using primers cel5A-fw (5′-TCC ATG GCA CAG CAG ACT GTC TGG GGC-3′) and cel5A-rv (5′-TGC GGC CGC CTT TCT TGC GAG ACA CG-3′), resulting in a product lacking the first 21 codons representing the fungal signal peptide. For constitutive expression, the PCR product was first transferred to vector pCR2.1 (Invitrogen, Darmstadt, Germany) by TA-cloning to generate pCR2.1-TrCel5A. Digestion with NcoI and NotI released a cassette which was then transferred to vector pTRAkc-AH [48] and digested with the same enzymes. For ethanol inducible expression [27], the *alc*Amin35s sequence was amplified from pTRAkt-alcR-alcA-stppc (kindly provided by HJ Hirsch, Institute of Botany, RWTH Aachen University, Germany) using primers alcAmin35s-fw (5′-AAG GAT CCA CCC GGG TGG CTA GAA ATA TTT GCG ACT CTT CTG-3′) and *alcA*min35s-rv (5′-AGC GGC CGC GTT TAA ACC AAT TGG TCC TCT CCA AAT GAA ATG AAC TTC C-3′). The MfeI-PmeI fragment was transferred to pTRAkc-AH-Cel5A linearized with EcoRI and PmeI, and was then subcloned as a BstXI-PmeI fragment into pTRAkt-alcR (kindly provided by HJ Hirsch, Institute of Botany, RWTH Aachen University, Germany) linearized with FseI and PmeI.

### Transgenic plants

The binary vectors described above were introduced into *Agrobacterium tumefaciens* strain GV3101::pMP90RK [49] by electroporation [50]. Transgenic tobacco lines were then generated (*N. tabacum* L. cv. Petit Havana SR1) using the leaf disc transformation method [34]. T_0_ plants were grown on Murashige-Skoog medium containing 100 mg/L kanamycin and 200 mg/L Claforan, and were subsequently transferred to soil in the greenhouse and selfed to produce the T_1_. T_1_ lines showing Mendelian segregation consistent with a single locus insertion were used to produce T_2_ generations which were used for all further assays.

### Ethanol induction

Induction of the ethanol switch was achieved by providing ethanol with the spilling water. Therefore, tobacco plants were treated once with 100 ml of a 2% (v/v) ethanol solution, this procedure was varied in time or dosage as described in the figure legends and results section. Induced plants were kept separately from non induced plants to avoid induction via ethanol vapor. As a control, wild-type (*N. tabacum* SR1) plants were treated equally and analyzed together with the transgenic plants.

### Protein extraction and purification

Transgenic leaves were ground in liquid nitrogen and homogenized in phosphate buffered saline (PBS; pH 7.0) supplemented with 1 mM phenylmethylsulfonyl fluoride (PMSF). The extract was centrifuged at 15,000 × *g* for 20 min at 4°C followed by filtration to remove particles. The His_6_-tagged protein was purified from TSP by Ni–NTA agarose affinity chromatography (Qiagen, Hilden, Germany). Imidazole was removed using a Roti®Spin column (Roth, Karlsruhe, Germany) with a MWCO of 10 kDa. Total protein levels were determined using the Bradford method [51] with bovine serum albumin (Roth, Karlsruhe, Germany) as the standard.

### SDS-PAGE, western blot and activity staining

Protein samples were separated by SDS-PAGE in a 12% polyacrylamide gel containing 0.15% (w/v) carboxymethylcellulose (CMC). The proteins were then renatured by washing twice at room temperature for 15 min with 50 mM potassium acetate buffer (pH 4.8) containing 20% (v/v) propan-2-ol followed by two 30-min washes in the same buffer without propan-2-ol. The samples were incubated in potassium acetate buffer at 50°C for 30 min and then in 50 mM Tris-HCl (pH 7.5) for 30 min at room temperature to stop the reaction. The gels were stained for 30 min in 0.1% (w/v) Congo Red (Sigma-Aldrich, Seelze, Germany) and destained in 1 M NaCl. The gel was incubated in 0.5% (v/v) acetic acid after destaining to increase the contrast.

For Western blot analysis, separated proteins were electro-transferred (60 min, 250 mA) to nitrocellulose membranes, blocked for 1 h at room temperature with 5% (w/v) skimmed milk in PBS, and then probed first with a polyclonal antibody recognizing *Trichoderma viride* cellulase (antibodies-online, Aachen, Germany) and second with a monoclonal alkaline phosphatase-conjugated goat anti-rabbit antibody (Dianova, Hamburg, Germany). The signal was visualized with nitroblue tetrazolium chloride/5-bromo-4-chloro-3′-indolyphosphate p-toluidine salt (NBT/BCIP) (Roth, Karlsruhe, Germany).

### Endoglucanase assays

Endoglucanase activity in crude plant extract was determined by the conversion of 4-methylumbelliferyl β-D-cellobioside (4-MUC) into 4MU, as described previously [12]. Samples were run in triplicate, with each sample (1–5 μl) assayed in 100 μl buffer (50 mM sodium acetate, pH 4.8, 0.5 mM 4-MUC) in a 96-well plate. Plates were covered with adhesive lids to prevent evaporation and incubated for 60 min at 50°C. The reaction was stopped by adding 100 μl 0.15 M glycine pH 10.0. The fluorescence was determined with Tecan Infinite M200 (excitation wavelength of 360 nm, emission wavelength 465 nm). Fluorescence data of TrCel5A activity, from induced and constitutively expressed plants, were corrected by subtraction of the average data from wild-type crude plant extract (*n*=3). Conversion rates were calculated from corrected data based on a series of 4MU standards (1–10 nM).

The soluble chromogenic substrate AZO-CM-Cellulose (Megazyme, Bray, Ireland) was used to determine the temperature and pH tolerance of the recombinant purified enzyme, as previously described [52], with five replicates taken at each measurement point. Temperature dependence was determined using 50 mM acetate buffer (pH 4.5) in the range 20–70°C. The pH tolerance was determined at 55°C using 50 mM citrate buffer (pH 2.0–3.5), 50 mM acetate buffer (pH 3.5–5.5) and 50 mM phosphate buffer (pH 5.5–7.0).

### Cellulose analysis

Cellulose was extracted from leaf tissue taken from 6–7 week-old plants. Ten samples were taken from each transgenic line and from wild-type tobacco plants. To determine the crystalline cellulose content, alcohol-insoluble residues (AIRs) were prepared as described by [36], by grinding 50 mg per sample to a fine powder under liquid nitrogen, and isolating the plant cell walls by washing with different organic solvents. Starch was removed by hydrolysis with amylase and pullulanase (Sigma-Aldrich). The remaining AIRs were extracted with acetone, dried and weighed. The crystalline cellulose content was determined according to established methods [37], after hydrolyzing the non-crystalline cellulose with acetic and nitric acids. The remaining crystalline cellulose residues were hydrolyzed with 72% sulfuric acid, allowing the remaining glucose to be measured using the anthrone assay [53]. Significant differences from wild-type cellulose content were determined using a Student *t*-test (p value ≤ 0.01).

## Abbreviations

4MU: 4-Methylumbelliferone; 4MUC: 4-Methylumbelliferyl β-D-cellobioside; AIR: Alcohol insoluble residues; BCIP: 5-Bromo-4-chloro-3′-indolyphosphate p-toluidine; CaMV: Cauliflower Mosaic Virus; CBM: Cellulose binding module; CMC: Carboxymethylcellulose; EG: Endoglucanase; ER: Endoplasmic reticulum; MAMP: Microbe-associated molecular pattern; NBT: Nitro-blue tetrazolium chloride; PASC: Phosphoric acid swollen cellulose; PBS: Phosphate buffered saline; PMSF: Phenyl-methylsulfonyl fluoride; SDS: Sodium dodecyl sulfate; TSP: Total soluble protein; Tris: Tris(hydroxymethyl)aminomethane; UTR: Untranslated region.

## Competing interests

The authors declare that they have no competing interests.

## Authors’ contribution

HK designed and carried out the experiments, analyzed results and wrote the manuscript. MG carried out the analysis of tobacco cell walls. BU assisted in the experimental design and reviewed the manuscript. RF and UC coordinated the study and reviewed the manuscript. All authors read and approved the final manuscript.

## Supplementary Material

Additional file 1 **Zymography performed with SDS-PAGE containing 0.15% (w/v) CMC (A) and Western blot (B) of transiently expressed TrCel5A.** The recombinant enzyme was detected in Western blot with monoclonal αanti-His antibody and alkaline phosphatase conjugated goat-anti-mouse secondary antibody. As indicated plant crude extract and purified TrCel5A were used. The upper band represents the holoenzyme the lower band represents the truncated enzyme containing the catalytic domain. Click here for file
