# Supplementary figures and images for: Ethanol inducible expression of a mesophilic cellulase avoids adverse effects on plant development
Source: Biotechnol Biofuels. 2013 Apr 16;6:53. doi: 10.1186/1754-6834-6-53 (PMC3643885; doi:10.1186/1754-6834-6-53)

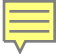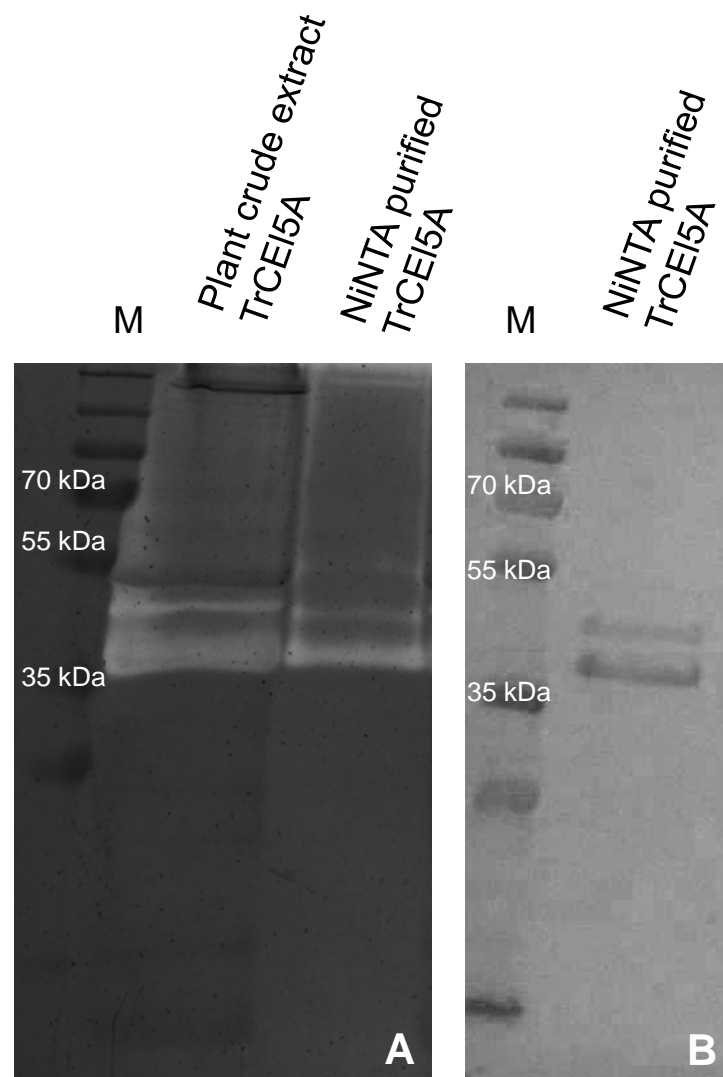

Supplement: Additional file 1 — Zymography performed with SDS-PAGE containing 0.15% (w/v) CMC (A) and Western blot (B) of transiently expressed TrCel5A. The recombinant enzyme was detected in Western blot with monoclonal αanti-His antibody and alkaline phosphatase conjugated goat-anti-mouse secondary antibody. As indicated plant crude extract and purified TrCel5A were used. The upper band represents the holoenzyme the lower band represents the truncated enzyme containing the catalytic domain. [file 1754-6834-6-53-S1.pdf]
